# Periostin-binding DNA aptamer treatment attenuates renal fibrosis under diabetic conditions

**DOI:** 10.1038/s41598-017-09238-6

**Published:** 2017-08-17

**Authors:** Jae Eun Um, Jung Tak Park, Bo Young Nam, Jung Pyo Lee, Jong Ha Jung, Youndong Kim, Seonghun Kim, Jimin Park, Meiyan Wu, Seung Hyeok Han, Tae-Hyun Yoo, Shin-Wook Kang

**Affiliations:** 1 Severance Biomedical Science Institute, College of Medicine, Yonsei University, Seoul, Korea; 2 Department of Internal Medicine, College of Medicine, Severance Biomedical Science Institute, Brain Korea 21 PLUS, Institute of Kidney Disease Research, Yonsei University, Seoul, Korea; 3 Department of Internal Medicine, Seoul National University Boramae Medical Center, Seoul, Korea; 4 Aptamer Sciences Inc., POSTECH Biotech Center, Pohang, Gyeongbuk Korea

## Abstract

Diabetic nephropathy, the major cause of chronic kidney disease, is associated with progressive renal fibrosis. Recently, accumulation of periostin, an extracellular matrix protein, was shown to augment renal fibrosis. Aptamers have higher binding affinities without developing the common side effects of antibodies. Thus, we evaluated the effect of periostin inhibition by an aptamer-based inhibitor on renal fibrosis under diabetic conditions. *In vitro*, transforming growth factor-β1 (TGF-β1) treatment significantly upregulated periostin, fibronectin, and type I collagen mRNA and protein expressions in inner medullary collecting duct (IMCD) cells. These increases were attenuated significantly in periostin-binding DNA aptamer (PA)-treated IMCD cells exposed to TGF-β1. *In vivo*, PA treatment attenuated the increased blood urea nitrogen levels in the diabetic mice significantly. Fibronectin and type I collagen mRNA and protein expressions increased significantly in the kidneys of diabetic mice: PA administration abrogated these increases significantly. Immunohistochemistry and Sirius Red staining also revealed that fibronectin expression was significantly higher and tubulointersititial fibrosis was significantly worse in diabetic mice kidneys compared with control mice. These changes were ameliorated by PA treatment. These findings suggested that inhibition of periostin using a DNA aptamer could be a potential therapeutic strategy against renal fibrosis in diabetic nephropathy.

## Introduction

Diabetic nephropathy is the leading cause of end-stage renal disease (ESRD) worldwide^1^. However, treatment strategies to prevent the deterioration of renal function in diabetic nephropathy patients are limited. The pathological features of diabetic nephropathy include glomerular and tubular hypertrophy, podocytopenia, and accumulation of extracellular matrix (ECM) proteins, such as collagen and fibronectin. ECM accumulation in diabetic nephropathy results in renal tubulointerstitial fibrosis, which causes irreversible renal dysfunction^2–4^.

Transforming growth factor (TGF)-β1 is regarded as one of the main mediators of the deleterious effects of high glucose in diabetic nephropathy^5^. TGF-β1 expression is increased in mesangial cells and tubular epithelial cells under diabetic conditions^6, 7^, and mediates profibrotic events, hypertrophy, and cell survival^8, 9^. In particular, renal tubulointerstitial fibrosis accompanied by diabetic nephropathy is largely attributed to the activation of this TGF-β1 pathway.

Periostin is an ECM protein that is upregulated by the activation of the TGF-β1 pathway in several cell types^10, 11^. It exhibits autocrine and paracrine functions by acting as a ligand of αVβ3 and αVβ5 integrins^12^, which induce transcription of ECM proteins, such as collagen and fibronectin, by activating the integrin-related intracellular pathway^13^. Previous studies showed that periostin was implicated in cardiac and dental development^14, 15^. In addition, the expression of periostin was demonstrated as crucial in wound healing, as well as in cancer cell metastasis, via promotion of epithelial-mesenchymal transition^15, 16^. Recent research also proposed a possibility that periostin might play a role in the pathogenesis of renal fibrosis^17^. The expression of periostin was increased in the kidneys of 5/6 nephrectomized mice and in unilateral ureter obstruction mouse models, and was correlated with renal fibrosis severity^18, 19^. Moreover, urinary periostin levels were increased significantly in diabetic patients with renal failure or albuminuria, suggesting a role of periostin in the progression of diabetic nephropathy.

Aptamers are oligonucleotides comprising DNA or RNA single strands that can bind to specific target proteins. Their ability to inhibit the activity of certain proteins through this protein-specific binding action has led to the application of aptamers in various clinical fields^20^. Aptamers are produced chemically and are relatively bio-stable, resulting in lower production costs, and more convenient storage and distribution compared with antibodies^20, 21^. Due to these advantages aptamers are considered as promising drug candidates in various diseases, such as HIV infection, macular degeneration, and diabetes^21–23^.

In this study, the inhibitory effect of a novel periostin-binding DNA aptamer (PA) on the activity of periostin on ECM accumulation under diabetic conditions was evaluated using cultured inner medullary collecting duct (IMCD) cells and mouse models of diabetes.

## Results

### TGF-β1 increases periostin expression and ECM synthesis in IMCD cells

**Figure 1:**
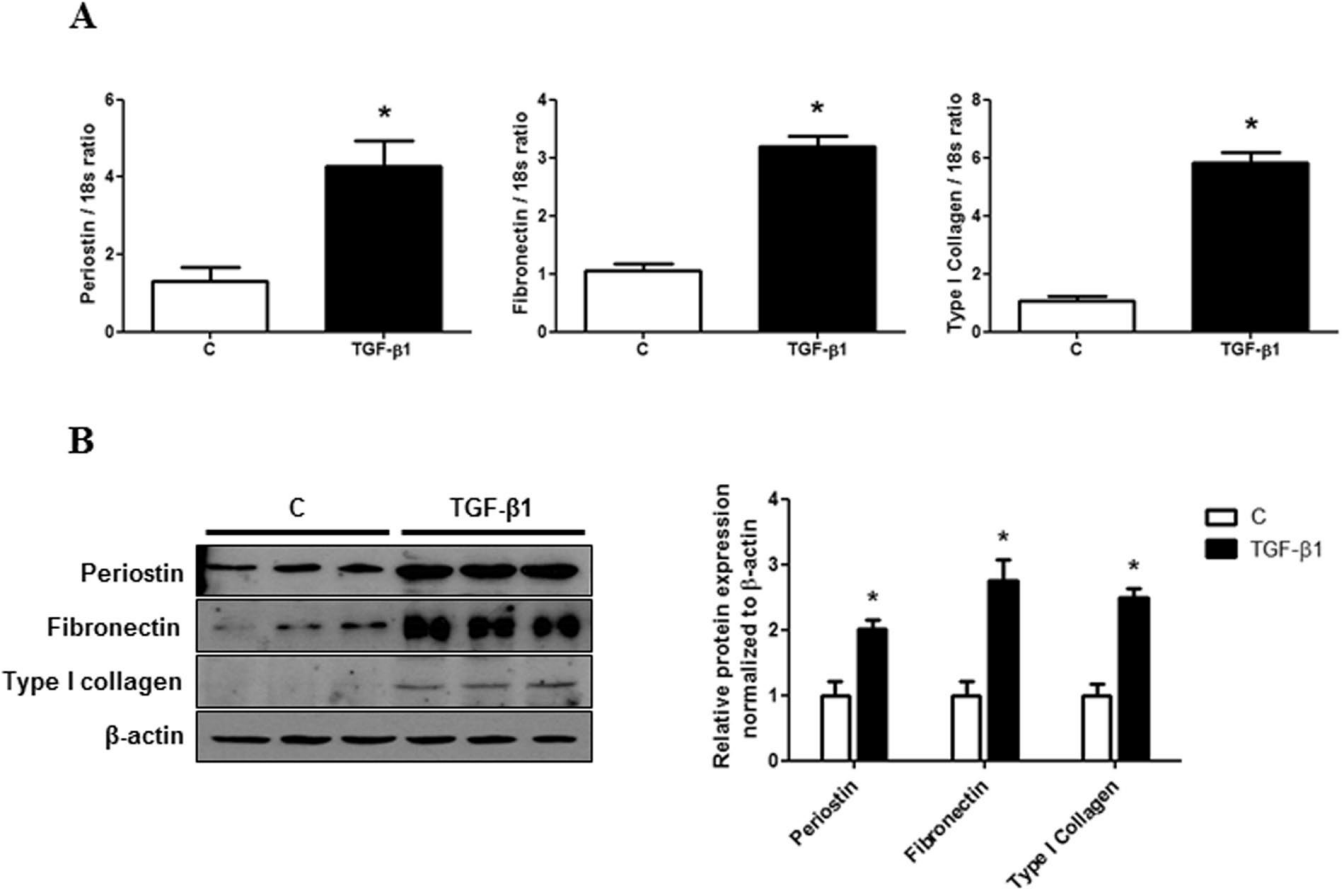
Periostin, fibronectin, and type I collagen expression in inner medullary collecting duct (IMCD) cells treated with or without TGF-β1. (**A**) Periostin, fibronectin, and type I collagen mRNA levels (n = 6). Compared to control IMCD cells, periostin, fibronectin, and type I collagen mRNA expression were significantly increased in TGF-β1-treated cells (10 ng/ml). (**B**) A representative western blot analysis of periostin, fibronectin, and type I collagen protein (a representative of 4 blots). The protein expression of periostin, fibronectin, and type I collagen were increased significantly in IMCD cells exposed to TGF-β1. Uncropped scans are presented in Supplementary Figure 2. C, control. *P < 0.05 vs. C.

First, to investigate whether periostin was associated with TGF-β1-induced renal fibrosis, the expression of periostin was evaluated in cultured IMCD cells treated with or without TGF-β1. Periostin mRNA and protein levels were increased significantly in IMCD cells exposed to TGF-β1 (10 ng/ml) compared with control cells (P < 0.01). This increase in periostin expression was accompanied by the upregulation of fibronectin and type I collagen mRNA and protein levels (P < 0.01) (Fig. 1). These results suggested that TGF-β1 induces ECM synthesis, together with a significant increase in periostin expression in IMCD cells.

### Periostin knockdown abrogates TGF-β1-induced ECM synthesis in IMCD cells

**Figure 2:**
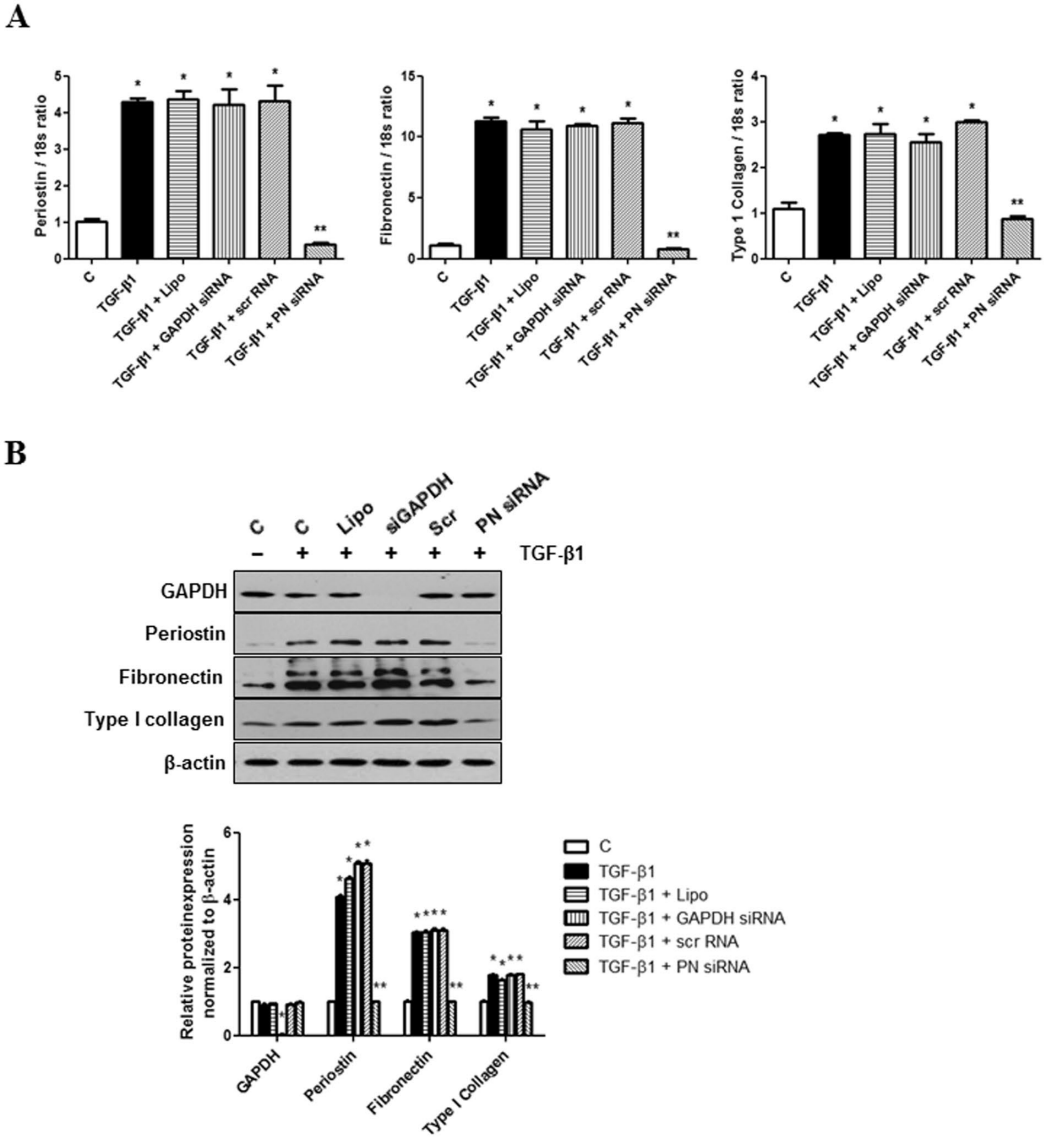
Changes in mRNA and protein expression of periostin, fibronectin, and type I collagen expression in inner medullary collecting duct (IMCD) cells following periostin siRNA transfection. (**A**) mRNA levels in TGF-β1-stimulated (10 ng/ml) IMCD cells treated with or without periostin siRNA (100 nM) (n = 12). Periostin siRNA significantly attenuated TGF-β1-induced increases in periostin, fibronectin, and type I collagen mRNA levels in IMCD cells treated with TGF-β1. Positive control GAPDH siRNA transfection significantly down-regulated GAPDH mRNA level. Negative control scramble siRNA transfection did not affect the expression of mRNA levels. (**B**) A representative western blot analysis of protein expression in TGF-β1-stimulated IMCD cells with or without periostin siRNA transfection (a representative of 4 blots). Periostin siRNA transfection significantly mitigated the increases in periostin, fibronectin, and type I collagen protein expression in TGF-β1-stimulated IMCD cells. Positive control GAPDH siRNA transfection significantly down-regulated GAPDH protein level. Negative control scramble siRNA transfection did not affect the expression of protein levels. Uncropped scans are presented in Supplementary Figure 3. C, control; Lipo, lipofectamine; siGAPDH, GAPDH siRNA; Scr, negative control scramble siRNA; PN siRNA, periostin siRNA. *P < 0.05 vs. C; **P < 0.05 vs. TGF-β1.

To evaluate the role of periostin in TGF-β1 induced ECM synthesis in IMCD cells, periostin expression was knocked down by siRNA transfection in TGF-β1-stimulated IMCD cells. As shown in Fig. 2A, periostin knockdown by siRNA administration effectively inhibited periostin mRNA expression induced by TGF-β1 treatment. The expression of fibronectin and type I collagen mRNA also significantly decreased in IMCD cells treated with TGF-β1 and periostin siRNA compared to IMCD cells treated with TGF-β1 alone. Regarding the protein levels, periostin siRNA significantly attenuated the increases in periostin, fibronectin, and type I collagen expression (Fig. 2B). Together, these results suggest that periostin up-regulation is an essential factor in ECM synthesis of TGF-β1-stimulated IMCD cells and that periostin inhibition can prevent the pathologic ECM accumulation in IMCD cells.

### Validation of DNA aptamer binding to periostin in IMCD cells

**Figure 3:**
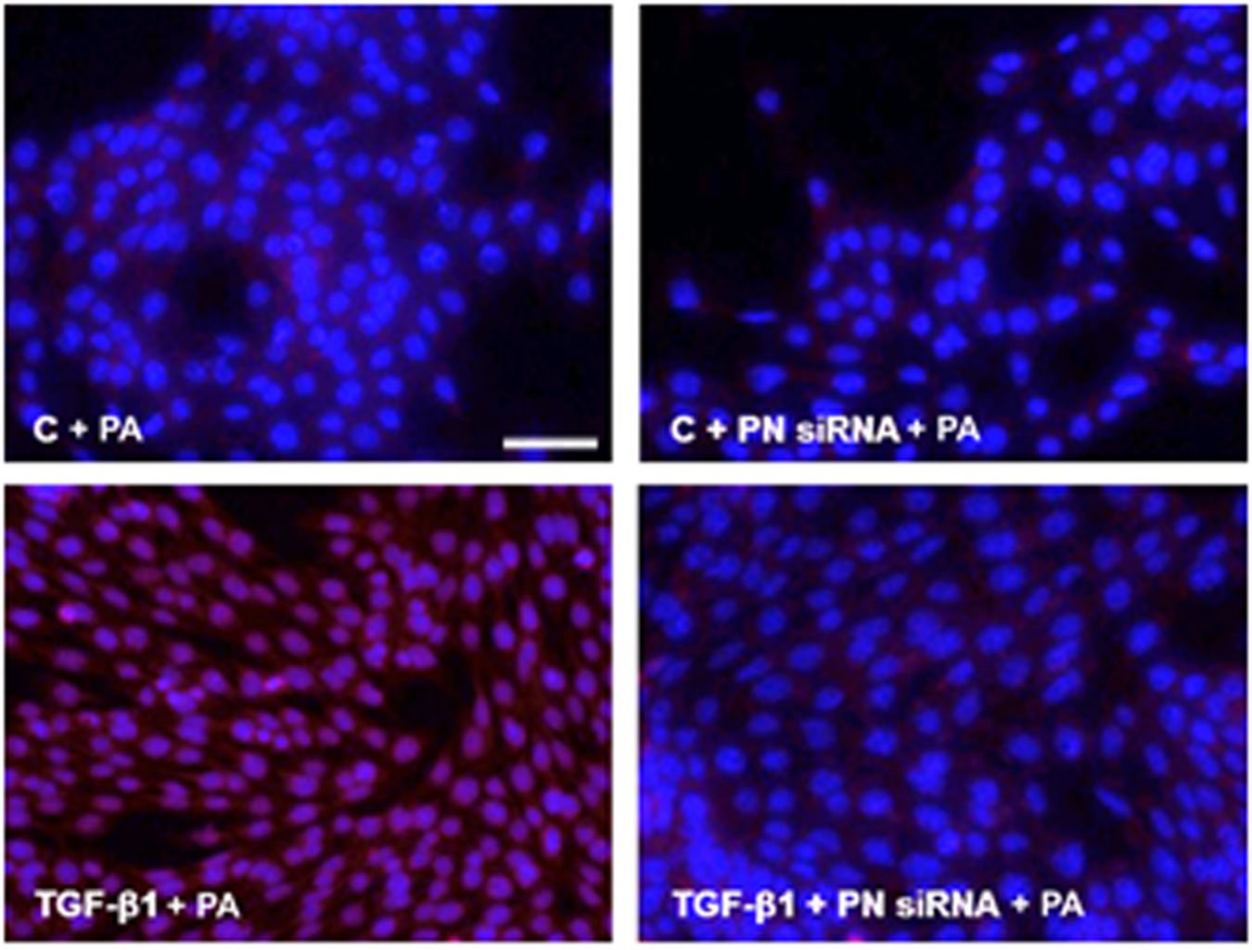
Specific attachment of the periostin-binding DNA aptamer (PA) to periostin expressed in inner medullary collecting duct (IMCD) cells. IMCD cells were treated with or without transforming growth factor-β1 (TGF-β1) (10 ng/ml), periostin siRNA (100 nM), or Cy3-tagged PA (100 pmol/ml). The intensity of Cy3 was significantly increased in TGF-β1-stimulated IMCD cells treated with the Cy3-tagged PA (scale bar, 50 μm). This effect was abrogated significantly by periostin siRNA transfection (×400). C, control; PN siRNA, periostin siRNA; PA, periostin-binding DNA aptamer.

To determine whether the PA attached specifically to periostin, the amount of a Cy3-tagged PA that was attached to periostin expressed in IMCD cells was evaluated. Compared with the negative control, the Cy3 intensity was increased significantly in the TGF-β1 (10 ng/ml)-stimulated IMCD cells treated with the Cy3-tagged PA (100 pmol/ml). This effect was attenuated significantly by transfection with a periostin siRNA (100 nM). These findings suggested that the PA binds specifically to periostin expressed in IMCD cells (Fig. 3).

### Periostin-binding DNA aptamer treatment ameliorates TGF-β1-induced ECM synthesis in IMCD cells

**Figure 4:**
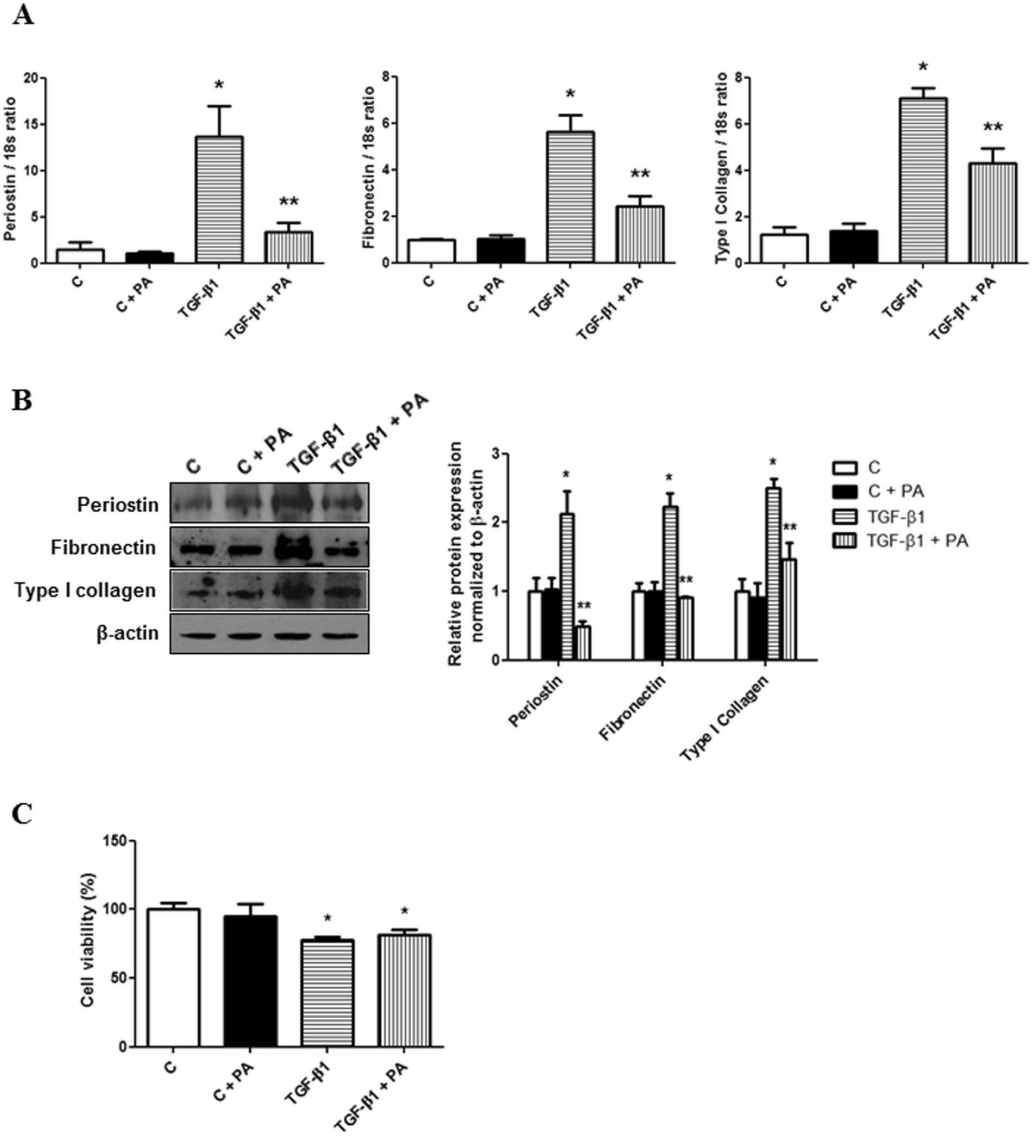
Changes in periostin, fibronectin, and type I collagen expression following periostin-binding DNA aptamer (PA) administration in inner medullary collecting duct (IMCD) cells treated with or without transforming growth factor-β1 (TGF-β1). (**A**) Periostin, fibronectin, and type I collagen mRNA levels (n = 6). Treatment with PA (100 pmol/ml) significantly ameliorated TGF-β1-induced (10 ng/ml) periostin, fibronectin, and type I collagen mRNA expression. (**B**) Representative western blotting analysis of periostin, fibronectin, and type I collagen protein levels (a representative of four blots). The increases in the protein levels of periostin, fibronectin, and type I collagen in TGF-β1-stimulated IMCD cells were significantly attenuated by the PA. Uncropped scans are presented in Supplementary Figure 4. (**C**) Cell viability in IMCD cells with or without treatment of PA and TGF-β1. Cell viability was not affected by PA treatment. However, cell viability was significantly decreased in TGF-β1-stimulated IMCD cells. C, control; PA, periostin-binding DNA aptamer. *P < 0.05 vs. C; **P < 0.05 vs. TGF-β1.

To evaluate whether inhibiting periostin signaling using the PA could affect ECM synthesis, the changes in fibronectin and type I collagen expression were explored after PA (100 pmol/ml) administration in TGF-β1 (10 ng/ml)-stimulated IMCD cells. The increases in periostin, fibronectin, and type I collagen mRNA expression were attenuated significantly by PA treatment in IMCD cells exposed to TGF-β1 (Fig. 4A). The increased protein levels of periostin, fibronectin, and collagen type I induced by TGF-ß1 stimulation were also abrogated significantly by PA treatment in IMCD cells (Fig. 4B). These results suggested that the PA could ameliorate the TGF-β1-induced ECM synthesis in IMCD cells effectively. The cell viability, which was assessed by methylthiazoletetrazolium (MTT) assay, decreased in TGF-β1 stimulated IMCD cells compared to control cells. However, the concentration of PA used in this study did not affect cell viability (Fig. 4C).

### Animal data

Next, to investigate the *in vivo* effect of PA treatment on renal fibrosis in diabetic nephropathy, the PA was injected into type I and type II diabetes mellitus (DM) mouse models intraperitoneally (500 µg/kg/d).

Type I DM mice were generated by injecting streptozotocin (STZ) into unilateral nephrectomized mice (UNXSTZ). Body weights were significantly lower in the UNXSTZ mice (23.5 ± 2.5 g) and UNXSTZ mice treated with the PA (UNXSTZ + PA; 21.5 ± 4.3 g) compared with those of the control mice (C; 28.2 ± 6.3 g) (P < 0.05). In contrast, the kidney weights were significantly higher in UNXSTZ (0.23 ± 0.05 g) and UNXSTZ + PA mice (0.19 ± 0.05 g) compared with those of the control mice (0.12 ± 0.04 g) (P < 0.05). The mean blood glucose concentrations were significantly higher in the UNXSTZ mice (576 ± 4.7 mg/dl) compared with those in the control mice (264 ± 4.9 mg/dl) (P < 0.01), which were not affected by PA treatment (587 ± 8.1 mg/dl). The levels of 24-h urinary albumin excretion were significantly higher in the UNXSTZ (0.21 ± 0.08 mg/day) mice than in the control mice (0.041 ± 0.006 mg/day) (P < 0.001), and the increase in urinary albumin excretion in the UNXSTZ mice was not changed significantly by treatment with the PA (0.19 ± 0.05 mg/day). However, the increase in blood urea nitrogen (BUN) concentrations in UNXSTZ mice (39.3 ± 2.4 mg/dl) was abrogated significantly by PA treatment (30.5 ± 4.0 mg/dl) (P < 0.05) (Table 1).

**Table 1:** Body weight, kidney weight, and 24-h urinary albumin excretion in type I diabetes mellitus (DM) mice.

	C (N = 8)	C + PA (N = 8)	UNXSTZ (N = 8)	UNXSTZ + PA (N = 8)
Blood glucose (mg/dL)	264 ± 4.9	267 ± 6.2	576 ± 4.7^*^	587 ± 8.1^*^
Body Wt (g)	28.2 ± 6.3	26.9 ± 7.5	23.5 ± 2.5^*^	21.5 ± 4.3^*^
Kidney Wt (g)	0.12 ± 0.04	0.11 ± 0.06	0.23 ± 0.05^*^	0.19 ± 0.05^*^
Urinary albumin excretion (mg/day)	0.04 ± 0.01	0.05 ± 0.01	0.21 ± 0.08^*^	0.19 ± 0.05^*^
BUN (mg/dL)	22.0 ± 3.8	21.7 ± 1.2	39.3 ± 2.4^*^	30.5 ± 4.0^**^

C, control; PA, periostin-binding DNA aptamer; UNXSTX, uninephrectomized STZ-induced DM mice; BUN, blood urea nitrogen. *P < 0.05 vs. C; **P < 0.05 vs. UNXSTX.

For the type II DM mouse model, *db/db* mice were used. Compared with control *db/m* mice, the changes in blood glucose concentrations, the kidney weight, and the 24-h urinary excretion levels in *db/db* mice were similar to those observed in the type I DM mouse model. Periostin-binding DNA aptamer treatment had no impact on blood glucose concentrations and the kidney weight in the *db/db* mice; however, the increase in 24-h urinary albumin excretion in *db/db* mice (0.59 ± 0.10 mg/day) was abrogated significantly by treatment with the PA (0.37 ± 0.11 mg/day) (P < 0.05). The increase in BUN levels in the *db/db* mice (29.3 ± 1.7 mg/dl) was also mitigated significantly by PA treatment (21.6 ± 5.0 mg/dl) (P < 0.05) (Table 2). In addition, the blood pressure was significantly higher in *db/db* mice compared to *db/m* mice. This was not significantly affected by PA treatment (Supplementary Figure 1).

**Table 2:** Body weight, kidney weight, and 24-h urinary albumin excretion in type II diabetes mellitus (DM) mice.

	*db/m* (N = 8)	*db/m* + PA (N = 8)	*db/db* (N = 8)	*db/db* + PA (N = 8)
Blood glucose (mg/dL)	264 ± 5.8	248 ± 4.8	555 ± 8.6^#^	610 ± 9.1^#^
Body Wt (g)	31 ± 1.2	32 ± 2.1	50 ± 6.5^#^	49 ± 4.2^#^
Kidney Wt (g)	0.20 ± 0.07	0.21 ± 0.06	0.25 ± 0.04^#^	0.26 ± 0.08^#^
Urinary albumin excretion (mg/day)	0.16 ± 0.06	0.19 ± 0.08	0.59 ± 0.10^#^	0.37 ± 0.11^†^
BUN (mg/dL)	22.2 ± 0.3	21.9 ± 0.8	29.3 ± 1.7^#^	21.6 ± 5.0^†^

C, control; PA, periostin-binding DNA aptamer; BUN, blood urea nitrogen. ^#^P < 0.05 vs. db/m; ^†^P < 0.05 vs. db/db.

### Periostin-binding DNA aptamer treatment ameliorates renal fibrosis in DM mouse models

**Figure 5:**
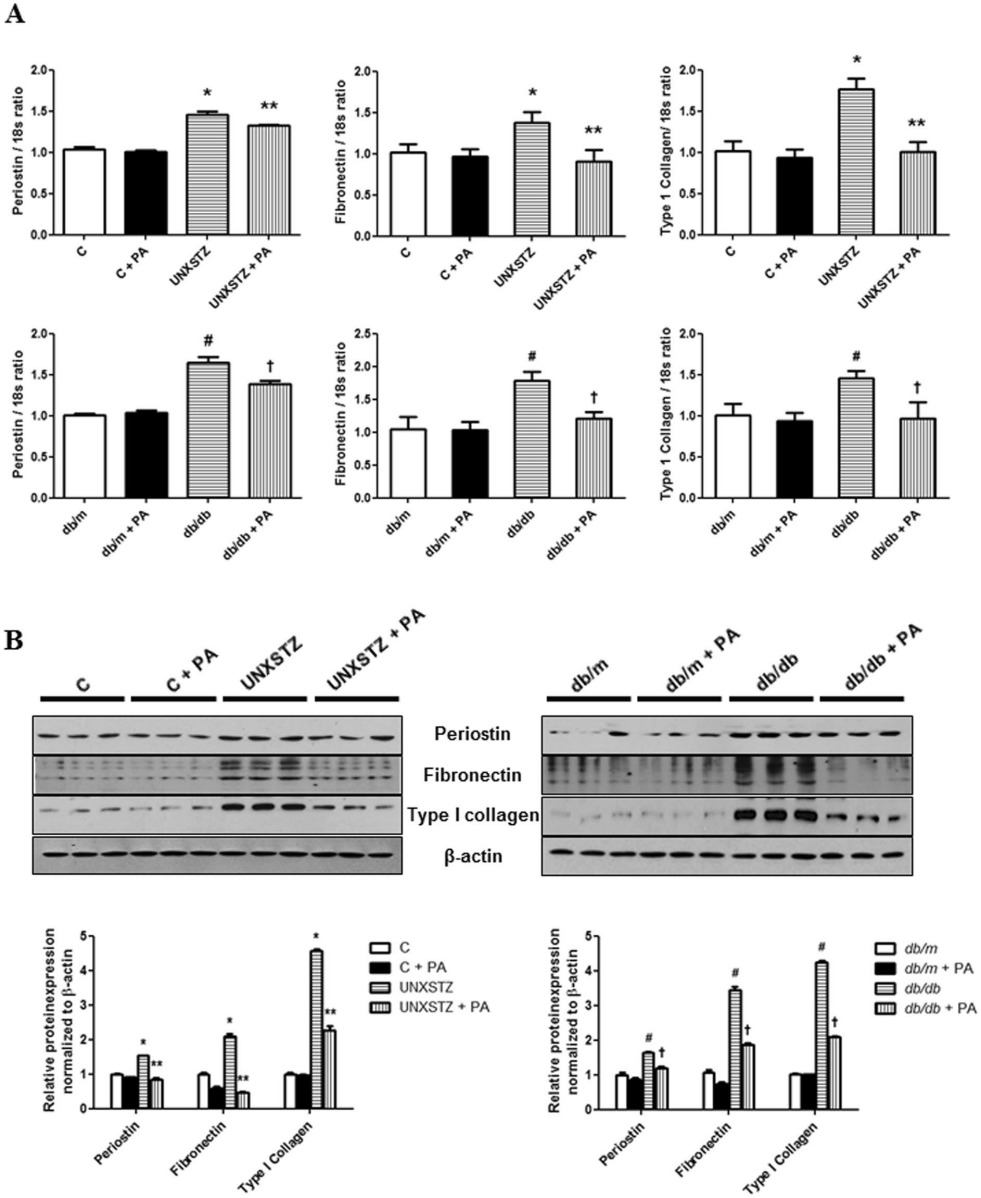
Renal periostin, fibronectin and type I collagen expression in diabetes mellitus (DM) and non-DM mice treated with or without a periostin-binding DNA aptamer (PA). (**A**) Periostin, fibronectin and type I collagen mRNA levels in the kidney (n = 8). Periostin, fibronectin and type I collagen mRNA expressions were significantly increased in uninephrectomized STZ-induced DM (UNXSTZ) and *db/db* mice compared with the control and *db/m* mice, respectively. These increases were significantly abrogated by PA administration (500 µg/kg/d). (**B**) Representative western blotting analysis of periostin, fibronectin, and type I collagen protein levels (a representative of four blots). The protein levels of periostin, fibronectin, and type I collagen were significantly increased in UNXSTZ and *db/db* mice compared with the control and *db/m* mice, respectively. The PA treatment significantly ameliorated these increases in the DM mice. Uncropped scans are presented in Supplementary Figure 5. C, control; PA, periostin-binding DNA aptamer; UNXSTX, uninephrectomized STZ-induced DM mice. *P < 0.05 vs. C; **P < 0.05 vs. UNXSTX; ^#^P < 0.05 vs. *db/m*; ^†^P < 0.05 vs. *db/db*.

**Figure 6:**
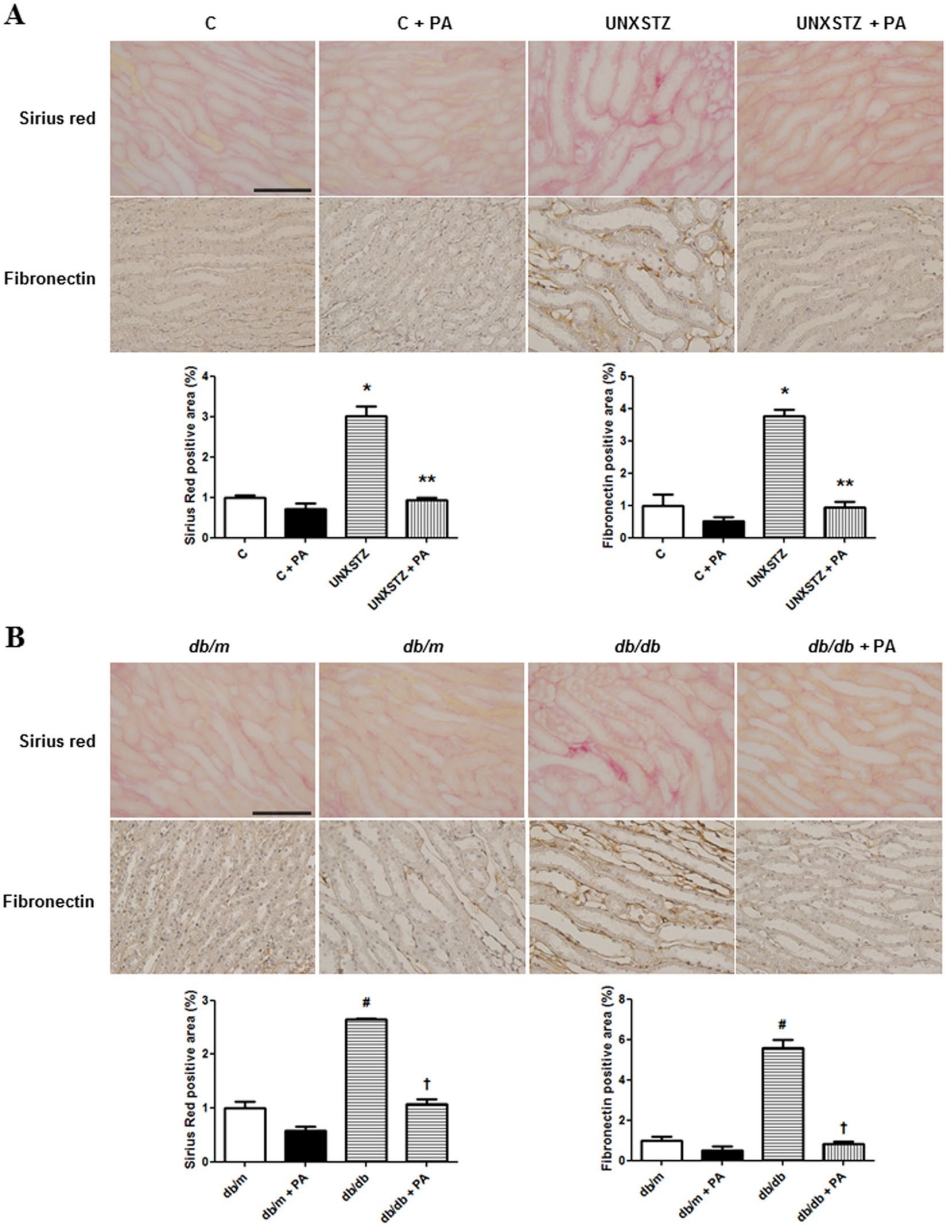
Immunohistochemical staining of fibronectin and Sirius Red staining in diabetes mellitus (DM) and non-DM mice treated with or without a periostin-binding DNA aptamer (PA). (**A**) Immunohistochemical staining and Sirius Red staining of the kidney revealed that renal fibronectin expression was significantly higher and renal fibrosis was more severe in uninephrectomized STZ-induced DM (UNXSTZ) compared with the control. (**B**) Renal fibronectin expression was significantly higher and renal fibrosis was more severe in *db/db* mice compared with *db/m* mice (scale bar, 50 μm). These changes in the DM mice were significantly attenuated by PA treatment (500 µg/kg/d) (×40). C, control; PA, periostin-binding DNA aptamer; UNXSTX, uninephrectomized STZ-induced DM mice. *P < 0.05 vs. C; **P < 0.05 vs. UNXSTX; ^#^P < 0.05 vs. *db/m*; ^†^P < 0.05 vs. *db/db*.

Periostin, fibronectin and type I collagen mRNA expression levels were significantly higher in the kidneys of type I and type II DM mice compared with those of control mice (P < 0.05). These increases in renal periostin, fibronectin and type I collagen mRNA expression in DM mice were attenuated significantly by PA treatment (Fig. 5A). The protein levels of periostin, fibronectin in the kidney, as assessed by western blotting, were also significantly higher in DM mice relative to control mice (P < 0.01). Periostin-binding DNA aptamer treatment abrogated these increases in DM mice significantly (Fig. 5B). Similarly, the staining intensity of fibronectin, as assessed by immunohistochemical staining, was significantly higher in the kidneys of DM mice, and was abrogated clearly by PA treatment. Sirius Red staining revealed that the renal fibrosis observed in the kidneys of DM mice was ameliorated significantly in PA treated DM mice (Fig. 6). Collectively, these findings indicated that the PA could attenuate effectively the renal fibrosis observed under diabetic conditions.

## Discussion

In the present study, periostin was observed to play a role in TGF-β1-induced ECM synthesis in cultured IMCD cells. Inhibition of periostin function by the PA resulted in the abrogation of TGF-β1-induced ECM accumulation in these cells. In addition, PA treatment inhibited tubulointerstitial fibrosis successfully in animal models of DM.

Tubulointerstitial fibrosis is one of the principal pathological features associated with renal function decline in patients with diabetic nephropathy^24^. A number of previous studies have suggested that tubulointerstitial lesions in diabetic patients are more useful to predict renal prognosis than glomerular or vascular lesions^25, 26^. Moreover, the severity of tubulointerstitial fibrosis correlated significantly with the degree of renal failure in patients with diabetic nephropathy^27^. The results of this study demonstrated the possibility of using PA treatment to delay the development of tubulointerstitial fibrosis and renal dysfunction in patients with diabetic nephropathy. The anti-fibrotic effect of inhibiting periostin in the current study agreed with the results of a recent investigation that evaluated the effect of transfecting antisense oligonucleotides against periostin on L-NAME (L-N^G^-Nitroarginine methyl ester)-induced renal injury model mice^18^. However, the fact that a novel aptamer-based approach was used in the present study to inhibit the development of diabetic nephropathy-associated tubulointerstitial fibrosis and renal failure should be considered. Furthermore, the advantages of aptamers over proteins or small-molecule-based treatment options, such as their low manufacturing cost and fewer side effects^28^, combined with the findings of this study, suggest aptamers as a practical and effective strategy for diabetic nephropathy treatment with potential in clinical applications.

Previously, interventions to regulate the deleterious effects of TGF-β1 have been proposed repeatedly in diabetic nephropathy. Administration of a neutralizing anti-TGF-β antibody was shown to significantly ameliorate renal ECM accumulation and to inhibit the decline in the glomerular filtration rate in diabetic animals^29^. In addition, substances having inhibitory effects on TGF-β1, such as decorin and pirfenidone [5-methyl-1-phenyl-2(1 H)-pyridone], have been tested for their efficacies in animal models of progressive renal disease^30–32^. Pirfenidone reduced proteinuria significantly and improved renal function in an anti-Thy-1 Ab model of progressive glomerulosclerosis^32^. In STZ-induced diabetic rats, pirfenidone also reversed the diabetes-induced renal deposition of both collagen and fibronectin^33^. However, in addition to promoting pathological fibrotic changes in disease states, TGF-β1 is also involved in various physiological functions, such as wound recovery and immune reactions^34, 35^. Moreover, TGF-β1 is located upstream of the TGF-β1-induced fibrosis signaling cascade; therefore, inhibition of TGF-β1 blocks numerous downstream cell signaling pathways that are critical for cell survival^36, 37^. For these reasons, concerns have been raised that chronic anti-TGF-β1 blockade might be deleterious because of its suppression of the immune system and loss of cell proliferation regulation^38^. Therefore, recent investigations have focused on molecular targets that are more downstream of the cell signaling cascade for anti-fibrotic effects. The fact that the inhibition of periostin, a terminally located protein of the TGF-β1-induced fibrosis pathway, attenuated tubulointerstitial ECM accumulation successfully in diabetic mouse models should be taken into account in this regard.

Treatment with a PA resulted in significant abrogation of renal ECM accumulation. Previous studies have demonstrated that periostin acts as a ligand and effects intracellular signaling through the activation of integrin αV/β3 and αV/β5^15^. The activation of integrin involves key cell signaling pathways that contribute to fibrosis, such as Akt and p38^39^. The periostin-specific binding property of the PA could inhibit the periostin-integrin interaction, thereby suppressing the integrin signaling pathways^40^. Further investigations of the cellular pathways that are affected by the PA are needed to confirm this notion. Furthermore, PA treatment resulted in downregulation of periostin expression in the present study. Periostin expression *per se* is regulated by integrin activation; therefore, it would seem logical that inhibiting the integrin activation function of periostin through the administration of a PA could down regulate the expression of periostin.

Albuminuria was reduced in type II DM *db/db* mice but not in type I UNXSTZ mice treated with the PA. In this study, the effect of inhibiting periostin function on ECM accumulation was evaluated only in the tubulointerstitial compartment because tubulointerstitial fibrosis is more evident in the course of renal disease progression compared to glomerular changes^41^. However, some studies found that periostin might also play a role in glomerular injury^16, 42, 43^. Biopsy samples from patients with glomerulopathies revealed an increased glomerular expression of periostin^16^. In addition, *in vitro* stimulation of mesangial cells with recombinant TGF-β1 resulted in significant induction of periostin^17^. Moreover, inhibition of periostin expression using antisense oligonucleotides ameliorated glomerulosclerosis in L-NAME-induced renal injury model mice^18^. The results of the present study showing that albuminuria was significantly reduced in PA treated-*db/db* mice might be attributed to improved glomerular pathology, partly caused by the inhibition of mesangial periostin function. However, further investigations to evaluate periostin expression within the glomeruli and the impact of inhibiting its function on diabetic glomeruli are needed to validate this assumption and to clarify the differential effect of the PA on albuminuria between type I and type II DM mice.

Although the results of this study have shown that PA treatment could inhibit renal tubular ECM accumulation in diabetic animals, translation to the clinical field should be done cautiously. Expression of periostin is known as a key event in skin healing, playing roles in the proliferative and remodeling phases of wound healing. Wound closure was found to be significantly delayed in periostin knockout mice compared to wild types, which was associated with reduction in wound contraction and re-epithelialization^44, 45^. Considering that wound healing delay is a common complication among diabetic patients, inhibition of periostin function through PA treatment may result in aggravated wound problems. Measures preventing skin complications should be preceded for the translational application of PA to diabetic patients.

In conclusion, the results of this study suggested that periostin acts as a key mediator of tubular fibrosis in DM patients, and treatment with the PA could be considered as a modality to prevent renal fibrosis under diabetic conditions. Nonetheless, clinical translation should be applied cautiously.

## Methods

### Periostin-binding DNA aptamer

Periostin-binding DNA aptamers were constructed by Aptamer Sciences Inc. (Pohang, South Korea), as previously reported^40^. Selected aptamers were prepared in two forms: a Cy3-labeled aptamer and a Polyethylene glycol (PEG)-conjugated aptamer. The Cy3-labeled periostin DNA aptamer was prepared by labeling with Cy3 at the 5′ end of the aptamer. The Cy3-labeled periostin DNA aptamer was used to evaluate the periostin-specific aptamer attachment in *in vitro* studies. PEG-conjugated PA was prepared by conjugating 40 kDa PEG to the 5′ end, and inverted dT to the 3′ end to increase its *in vivo* biological stability. PEG-conjugated PA was applied in the animal studies.

### Cell culture and treatment of IMCD cells

IMCD cells were maintained in DMEM/F12 medium supplemented with 10% fetal bovine serum (FBS), 100 U/ml penicillin, 100 mg/ml streptomycin, and 26 mM NaHCO_3_, and grown at 37 °C in humidified 5% CO_2_ in air. The medium was replaced by DMEM/F12 medium containing 5.6 mM glucose at 48 h after seeding. Subconfluent IMCD cells were FBS-restricted for 24 h, after which the medium was exchanged into 0.5% FBS DMEM/F12 medium for the control group and the same medium with TGF-β1 (10 ng/ml) (R&D Systems, Minneapolis, MN, USA) for the intervention group, with or without the PA (100 pmol/ml). IMCD cells were harvested for RNA and protein analyses at 48 h after media change.

### Animal experiments

The protocols for the animal experiments were approved by the Committee for the Care and Use of Laboratory Animals at Yonsei University College of Medicine in Seoul, Korea. All of the animal experiments were conducted in accordance with the Principles of Laboratory Animal Care (NIH Publication no. 85–23, revised 1985).

Thirty-two male C57BL/6 mice weighing 20–25 g were used. Unilateral nephrectomy (UNX) was performed, and one week after UNX, the mice were injected with 50 mg/kg of STZ (Sigma-Aldrich, St. Louis, MO, USA) intraperitoneally for 5 consecutive days (diabetes, DM). The control mice were injected with diluent. Each group consisted of 16 mice. Tail vein blood glucose levels were measured to confirm diabetes (fasting blood glucose > 300 mg/dL). The PA (500 µg/kg/d) was injected intraperitoneally daily in eight mice from each group. The mice were sacrificed at 7 weeks post-induction of diabetes.

Type 2 DM db/db mice and genetic control non-DM db/m mice (10–12 weeks old, 16 per group) were obtained from the Jackson Laboratories (Bar Harbor, ME, USA). The PA (500 µg/kg/d) was injected intraperitoneally daily in eight mice from each group, and the mice were sacrificed after 9 weeks.

Body weight, kidney weight, blood glucose levels, and 24-h urinary albumin excretion were determined at the time of sacrifice. Blood glucose was measured using a glucometer, and 24-h urinary albumin excretion was assessed by an enzyme-linked immunosorbent assay (ELISA) (Nephrat II, Exocell, Inc., Philadelphia, PA, USA). Blood pressure was measured by a noninvasive approach by a validated tail-cuff system using volume pressure recording technology (BP-2000, Visitech Systems, Apex, NC, USA). The mice were adapted to caging in the measuring chamber prior to measurement. After the initial acclimatization of five cycles, blood pressure was measured for 10 cycles and the average level was derived.

### IMCD cell transfection

For periostin knockdown experiments, periostin siRNA and negative control scramble siRNA was purchased from Dharmacon (Lafayette, CO, USA). Positive control GAPDH siRNA was purchased from Bioneer (Daejeon, South Korea). Periostin siRNA was transfected with Lipofectamine 2000 (Invitrogen, Carlsbad, CA, USA) according to the manufacturer’s protocol. Briefly, 6 μl of Lipofectamine 2000 was diluted in 1 ml of Opti-MEM I Reduced Serum Medium (Invitrogen), incubated for 15 min at room temperature (RT), and mixed with periostin siRNA (100 nM). After 15-min incubation at RT, the mixture was added to each well containing IMCD cells, which were plated at a density of 5 × 105 cells/well into six-well plates the day before, and the medium was changed after 24 h. The doses of TGF-β1 and periostin siRNA for this study were determined based on preliminary experimental results.

### Methylthiazoletetrazolium assay

To examine the cytotoxicity of the agents used, IMCD cells were cultured in 96-well culture plates, phenol red-free DMEM with 1 mg/ml of MTT were added to each well after the experimental periods, and then incubated for 2 h at 37 °C in humidified 5% CO2. After the incubation, extraction buffer (20% SDS, 50% N,N-dimethylformamide, pH 4.7) was added, following an overnight incubation at 37 °C. Optical density (OD) was measured with a microplate reader (SpectraMax 340, Molecular Devices) at a wavelength of 562 nm. The OD of the control group cells was assigned a relative value of 100. The experiments were performed in triplicates.

### Quantitative real-time polymerase chain reaction (qPCR)

The RNA extraction methods from IMCD cells and kidney samples were as described in a previous study^46^. A cDNA synthesis kit (Boehringer Mannheim GmbH, Mannheim, Germany) was used to obtain first-strand cDNA. The reactions were run on an ABI PRISM 7700 Sequence Detection System (Applied Biosystems, Foster City, CA, USA), and comprised a total volume of 20 µL containing 10 µL of SYBR Green PCR Master Mix (Applied Biosystems), 5 µL of cDNA, and 5 pmol sense and antisense primers. The primers used for periostin, fibronectin, type I collagen, and 18 s amplification were as follows: periostin, sense 5′-GGCACCAAAAAGAAATACT-3′ antisense 5′-GGAAGGTAAGAGTATAT-3′; fibronectin, sense 5′-TGACAACTGCCGTAGACCTGG-3′, antisense 5′-TACTGGTTGTAGGTGTGGCCG-3′; type I collagen, sense 5′-ACTGGTACATCAGCCCGAAC-3′, antisense 5′-TACTCGAACGGGAATCCATC-3′; and 18s, sense 5′-AACTAAGAACGGCCATGCAC-3′, antisense 5′-CCTGCGGCTTAATTTGACTC-3′. The RNAs used for amplification were 25 ng per reaction tube. The primer concentrations were determined by preliminary experiments that analyzed the optimal concentrations of each primer. The PCR conditions were as follows: 35 cycles of denaturation for 30 min at 94.5 °C, annealing for 30 sec at 60 °C, and extension for 1 min at 72 °C. Initial heating for 9 min at 95 °C and final extension for 7 min at 72 °C were performed for all PCR reactions. Each sample was run in triplicate in separate tubes and a control without cDNA was also run in parallel with each assay. After real-time PCR, the temperature was increased from 60 to 95 °C at a rate of 2 °C/min to construct a melting curve. The cDNA content of each specimen was determined using the comparative cycle threshold (CT) method with 2^−ΔΔ^CT. The results were given as the relative expression normalized to the expression of 18S rRNA and expressed in arbitrary units.

### Western blot analysis

Western blotting was performed as described previously^46^. Blots were incubated overnight at 4 °C with polyclonal antibodies to periostin (Abcam, Cambridge, UK), fibronectin (Dako, Glostrup, Denmark), type I collagen (Southern Biotech, Birmingham, AL, USA), or β-actin (Sigma Chemical Co., Perth, Australia). The membranes were washed three times for 10 min in 1 × PBS with 0.1% Tween-20 and incubated in buffer A containing a 1:1000 dilution of horseradish peroxidase-linked goat anti-rabbit or anti-mouse IgG (Santa Cruz Biotechnology, Inc., Santa Cruz, CA, USA). TINA image software (Raytest, Straubenhardt, Germany) was used to measure the band densities, and the changes in the optical densities of bands from the treated groups relative to control cells or tissues were used for analysis.

### Immunohistochemical staining and Sirius Red staining

Peritoneum sample fixation was done by using 10% neutral-buffered formalin. Paraffin-embedded tissues were processed in 5 µm-thick sections for immunohistochemical staining. Tissue sections were deparaffinized, rehydrated in ethyl alcohol, and washed in tap water. Antigen retrieval was performed in 10 mM sodium citrate buffer for 20 min using a Black & Decker vegetable steamer. Slides were blocked with 10% donkey serum for 30 min at room temperature and then washed with PBS. Primary antibodies for fibronectin was diluted to the appropriate concentrations with 2% casein in bovine serum albumin, added to the slides, and then incubated overnight at 4 °C. After washing, the slides were treated with a secondary antibody for 1 h at room temperature. Diaminobenzidine was added for 2 min, and hematoxylin was used to counterstain the slides. For Sirius Red staining, paraffin-embedded tissues processed in 5 µm-thick sections were deparaffinized, rehydrated in ethyl alcohol, washed in tap water, and re-fixed in Bouin’s solution for 1 h at 56 °C. After rinsing in running tap water for 10 min and staining with Weigert’s iron hematoxylin working solution for 10 min, the slides were stained with Biebrich scarlet-acid fuchsin solution for 15 min and washed in tap water. The sections were differentiated in phosphomolybdic-phosphotungstic acid solution for 15 min, transferred to aniline blue solution, and stained for 10 min. After rinsing briefly in tap water, the slides were reacted with 1% acetic acid solution for 5 min. A semi-quantitative score for the staining intensity was assessed by examining at least 10 fields in each section under ×400 magnification using digital image analysis (MetaMorph version 4.6r5, Universal Imaging Corp., Downingtown, PA, USA).

### Statistical analysis

Statistical analyses were conducted using SPSS for Windows version 21 (IBM Corporation, Armonk, NY, USA). Data are expressed as means ± standard errors of the mean (S.E.). The results were analyzed using the Kruskal-Wallis nonparametric test for multiple comparisons. Significant differences by the Kruskal-Wallis test were confirmed by the Mann-Whitney U-test. A P-value less than 0.05 was considered statistically significant.

## Electronic supplementary material

Supplementary information
