# Supplementary material for: Periostin-binding DNA aptamer treatment attenuates renal fibrosis under diabetic conditions
Source: Sci Rep. 2017 Aug 17;7:8490. doi: 10.1038/s41598-017-09238-6 (PMC5561139; doi:10.1038/s41598-017-09238-6)
Supplement: Supplementary file 1 — Supplementary information [file 41598_2017_9238_MOESM1_ESM.pdf]

Supplementary Information

Periostin-binding DNA aptamer treatment attenuates renal fibrosis under diabetic conditions

Jae Eun Um[1], Jung Tak Park[2*], Bo Young Nam[1], Jung Pyo Lee[3], Jong Ha Jung[4], Youndong Kim[4], Seonghun Kim[2], Jimin Park[2], Meiyan Wu[2], Seung Hyeok Han[2], Tae-Hyun Yoo[2], and Shin-Wook Kang[2]*

[1]Severance Biomedical Science Institute, College of Medicine, Yonsei University, Seoul, Korea, [2]Department of Internal Medicine, College of Medicine, Severance Biomedical Science Institute, Brain Korea 21 PLUS, Institute of Kidney Disease Research, Yonsei University, Seoul, Korea, [3]Department of Internal Medicine, Seoul National University Boramae Medical Center, Seoul, Korea, [4]Aptamer Sciences Inc., POSTECH Biotech Center, Pohang, Gyeongbuk, Korea

Correspondence to:

Shin-Wook Kang, MD, PhD

Yonsei University College of Medicine, Department of Internal Medicine

134 Shinchon-Dong, Seodaemoon-Gu, Seoul, Korea, 120-752; Phone 82-2-361-5428; FAX 82-2-393-6884; kswkidney@yuhs.ac

Supplementary Figure 1

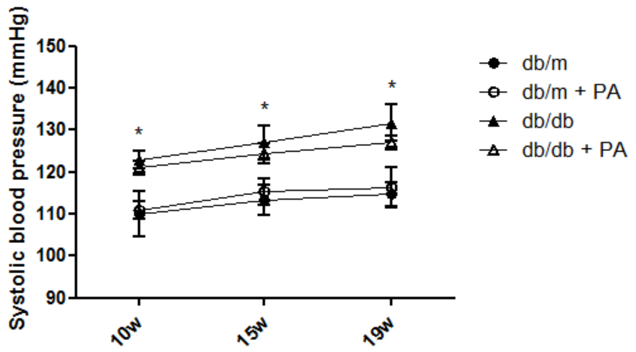

Supplementary Figure 1. Effect of periostin-binding DNA aptamer (PA) (500 µg/kg/d) on blood pressure in db/db and db/m mice. Time course of systolic blood pressure changes of *db/db* and *db/m* mouse groups. The systolic blood pressure was significantly elevated in *db/db* mice compared to *db/m* mice at 10, 15, and 19 weeks of age. The treatment of PA did not have a significantly effect on blood pressure in both *db/db* and *db/m* mice (n = 6 each group).

PA, periostin-binding DNA aptamer; W, weeks. *, $P < 0.05$ vs. *db/m*

Supplementary Figure 2

Fig.1B

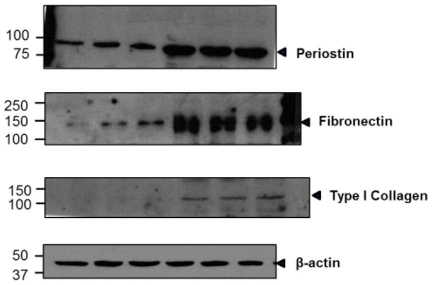

Supplementary Figure 2. Wider (uncropped) scans of blots (Figure 1B).

Supplementary Figure 3

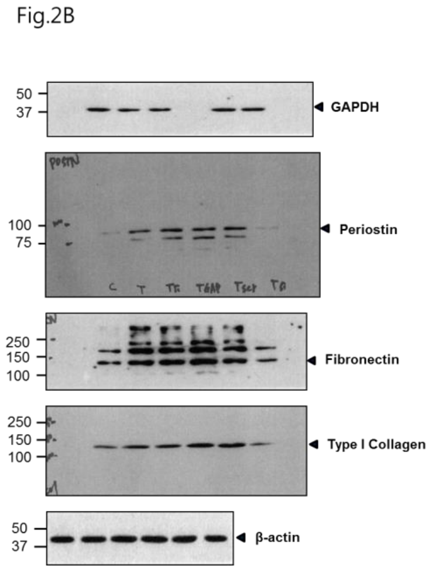

Supplementary Figure 2. Wider (uncropped) scans of blots (Figure 2B).

Supplementary Figure 4

Fig.4B

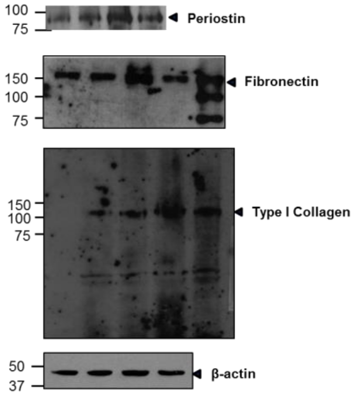

Supplementary Figure 3. Wider (uncropped) scans of blots (Figure 4B).

Supplementary Figure 5

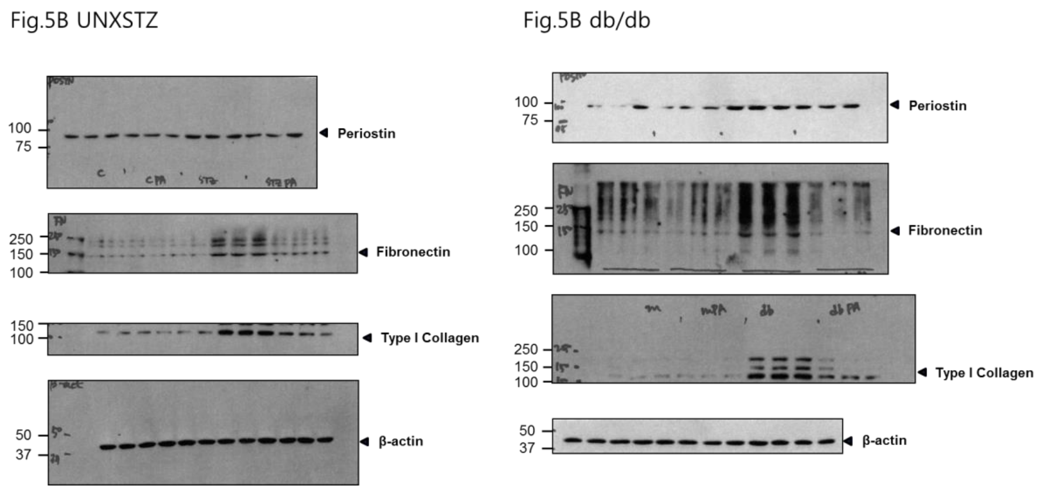

Supplementary Figure 4. Wider (uncropped) scans of blots (Figure 5B).
